# Pathobiology of aging and age-related diseases is the official journal of the Geropathology Research Network

**DOI:** 10.1080/20010001.2019.1593786

**Published:** 2019-03-18

**Authors:** Warren Ladiges

**Affiliations:** Department of Comparative Medicine, University of Washington, Seattle, WA, USA

We are pleased to announce that Pathobiology of Aging and Age-related Diseases is now the official journal of the Geropathology Research Network (GRN). In this capacity, the GRN will use the journal to publish workshop and conference proceedings, as well as technical articles related to pathological assessment of age-related lesions in rodents, other laboratory animals, pet dogs and cats, other domestic animals, nonhuman primates, and humans. The GRN has an active working committee, designated as the Geropathology Grading Committee, focusing on the establishment of guidelines for grading severity of lesions in tissues collected from mice in genetic or pharmacological aging intervention studies. In addition, Lesion of the Month and Geropathology in practice sections will be added to the journal. It is anticipated that these additions will be of interest, and help generate comments and feedback, as well as provide an increased awareness of the critical role geropathology plays in aging research, especially in preclinical studies, but in clinical studies as well.

In addition to an active lesion grading committee, the GRN has an active molecular pathology working group focusing on identifying and characterizing secretory proteins associated with aging and age-related diseases, especially neurological. The group, designated as the Geropathology Secretome Committee, will be contributing technical as well as scientific articles for publication.

Lastly, as the result of extensive tissue collection from rodents in aging studies in the last several years, the GRN has established a Geropathology Rodent Tissue Bank. This is just now getting started and will be ready for requests in the coming months from the scientific community. The journal will publish links for the tissue bank database, and periodic updates for issues of interest.

We look forward to these relevant and interesting contributions.

